# Emerging Applications of Aryl Trifluoromethyl Diazoalkanes and Diazirines in Synthetic Transformations

**DOI:** 10.1021/acsorginorgau.1c00027

**Published:** 2022-01-08

**Authors:** Thierry Ollevier, Virginie Carreras

**Affiliations:** Département de chimie, Université Laval, 1045 avenue de la Médecine, Québec, Québec G1V 0A6, Canada

**Keywords:** diazoalkane, diazirine, fluorine, trifluoromethyl group, carbene, cycloaddition, insertion, coupling

## Abstract

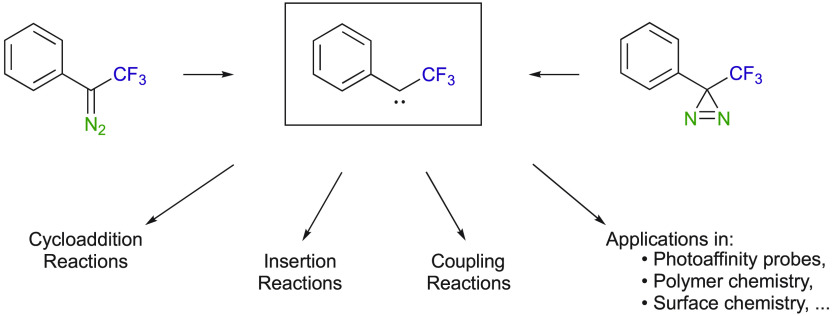

Aryl trifluoromethyl diazoalkanes and diazirines have become unique as reactants in synthetic methodology. As privileged compounds containing CF_3_ groups and ease of synthetic access, aryl trifluoromethyl diazoalkanes and diazirines have been highlighted for their versatility in applications toward a wide range of synthetic transformations. This Perspective highlights the synthetic applications of these reactants as precursors of stabilized metal carbenes, i.e., donor–acceptor-substituted ones.

## Introduction

Diazoalkanes have been investigated as building blocks in synthetic chemistry, and they have been extensively used in recent years.^1−3^ In particular, the features of metal carbenes derived from diazoalkanes substituted with a donor and an acceptor group have demonstrated their higher stability and selectivity.^4,5^

While CF_3_ groups have gained considerable importance and have been widely used in many fields, aryl trifluoromethyl diazo compounds have attracted limited attention. Indeed, trifluoromethyl diazo compounds have not been as exploited as diazo esters. In fact, the introduction of a CF_3_ group has a considerable impact on both the steric and electronic properties of the compounds, mainly due to its steric hindrance as the CF_3_ group is similar in size to the isopropyl group. This size has a direct impact on the hydrophobic character of a bioactive molecule. According to the Pauling scale, fluorine is the most electronegative element, which explains the strong inductive attracting characteristic of the CF_3_ group.^6^ The p*K*_a_ and lipophilicity of a compound can also be modified by the introduction of a CF_3_ group. Its presence within a bioactive molecule modifies its physical and chemical properties, having significant consequences on its biological activity. Today about 20–30% of pharmaceuticals and agrochemicals contain fluorine. The CF_3_ group is also used for its properties in chemical biology and materials chemistry.

Diazoalkanes and diazirines are well-known carbene precursors and are highly versatile compounds in organic synthesis.^2,7−9^ They are efficient compounds for creating carbon–carbon and carbon–heteroatom bonds that would be difficult to reach otherwise.^5^ As a result, several reviews have comprehensively reported different aspects of diazoalkane chemistry in the synthesis of organofluorine compounds.^10,11^ Moreover, the chemistry of 2,2,2-trifluorodiazoethane (CF_3_CHN_2_) has already been covered in a detailed and inclusive review.^12^ In the interest of brevity and to avoid discussing this reagent once again, this Perspective will focus on disubstituted diazoalkanes and diazirines as aryl trifluoromethyl precursors of carbene intermediates, as donor–acceptor-substituted ones.^5,13^ As such, this Perspective will highlight recent synthetic developments using aryl trifluoromethyl diazo and diazirine compounds that have been reported since 2011 until mid-2021. Aryl trifluoromethyl diazolkanes and diazirine are privileged compounds containing the CF_3_ group and are easy to prepare; their versatility has been demonstrated in a wide range of synthetic transformations. Diazoalkanes and diazirines can be decomposed by a photochemical or heating process to generate free carbenes. Decomposition of diazoalkanes can also be achieved using metals to give metal carbenes.^14,15^ The stability of the metal carbene increases with mesomeric stabilization with acceptor substituents, such as esters, and decreases with electron-donor groups, such as alkyl and aryl groups, giving donor–acceptor carbenes much greater selectivity.^5,16^

**Scheme 1:**
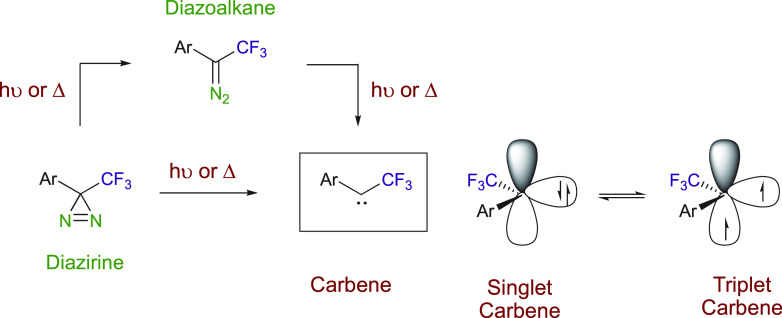
Generation of Carbene Intermediates from Diazoalkanes and Diazirines

Both diazo compounds and diazirines have been used as carbene sources (Scheme 1). Whereas diazo compounds have been extensively used in synthetic organic chemistry,^3,8^ diazirines have been mostly employed as photoaffinity reagents to label receptors and linking reagents.^9,17−24^ Numerous applications in medicinal chemistry (i.e., as photoaffinity probes),^21,24−29^ surface chemistry (i.e., for analyte immobilization),^30,31^ and polymer cross-linking^18,32^ have been found with trifluoromethyl diazirines. Yet, the chemistry of diazirines has been much less developed in synthetic chemistry than the that of diazoalkanes. Therefore, their use in organic chemistry needs to be better known and understood. Importantly, compared to that of aryl trifluoromethyl diazoalkanes, aryl trifluoromethyl diazirines exhibit higher stability in acidic or basic conditions, less toxicity, and higher bench stability.^20,33−35^ The synthesis of aryl trifluoromethyl diazirines usually involves the preparation of a tosyl oxime from an aryl trifluoromethyl ketone, followed by the treatment with liquid ammonia to give the corresponding diaziridine.^20^ Next, the oxidation of the diaziridine into the diazirine was reported using various oxidants, such as Ag_2_O, KMnO_4_, or I_2_.^20,36−38^ Upon photochemical of thermal treatment, diazirines lose nitrogen to form carbenes, either directly or through isomerization to the diazoalkane isomer.^39^ A wide range of reactions can then be instigated depending on the spin state of the carbene.

Although aryl trifluoromethyl diazo and diazirine compounds have been less extensively studied in the past^40−42^ than diazoalkanes and diazirines substituted with an aryl and an ester,^4,5,8^ the number of publications describing challenging synthetic methods using this class of reactant is now rapidly growing. Aryl trifluoromethyl diazo and diazirine compounds have been used in various types of reactions, ranging from cycloaddition to insertion and coupling reactions. This Perspective presents the use of these diazoalkanes and diazirines throughout the various types of reaction categories. Importantly, diazirine reactivity will be discussed in parallel in the same sections to establish a better comparison with diazoalkanes. With the present Perspective, we aim to inspire researchers to explore new routes and take advantage of the versatility of such compounds.

One of the major synthetic applications of diazoalkanes and diazirines is efficient carbon–carbon and carbon–heteroatom bond formation toward various carbocycles and heterocycles.

## Cycloadditions

[2 + 1] Cycloadditions are important synthetic transformations giving access to CF_3_-substituted cyclopropanes and cyclopropenes, including enantioselective versions. Other types of cycloadditions, particularly in metal-catalyzed multicomponent reactions, have also emerged in the literature. A number of recent cycloadditions involving the use of light-emitting diodes (LEDs) in flow chemistry is noteworthy.

### [2 + 1] Cycloadditions

#### Cyclopropanations

**Scheme 2:**
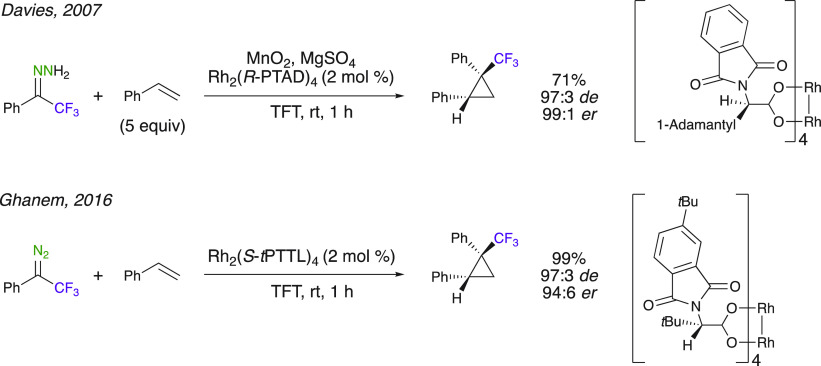
Asymmetric [Rh_2_(*S*-PTAD)_4_]- and [Rh_2_(*S*-*tert*PTTL)_4_]-Catalyzed Cyclopropanation Reaction of 2,2,2-Trifluoromethyl-1-phenyldiazoethane with Styrene

Whereas asymmetric cyclopropanation reactions have been extensively reported with various diazoalkanes using various metal catalysts, the use of aryl trifluoromethyl-substituted diazoalkanes remained limited until the 2000s. Aryl trifluoromethyl-substituted diazo compounds are now commonly used as carbene precursors in cyclopropanations. The asymmetric synthesis of trifluoromethyl-substituted cyclopropanes was developed by Davies et al. using chiral Rh^II^ complexes (Scheme 2).^40^ In situ formation of the diazo compounds was described from the oxidation of the corresponding trifluoromethyl-substituted hydrazone using MnO_2_. Interestingly, the oxidation conditions did not interfere with the subsequent enantioselective step. This in situ generation is a major asset as some of these trifluoromethyl-substituted diazo compounds are volatile and cannot be easily isolated. Excess of styrene, together with the use of α,α,α-trifluorotoluene (TFT) as solvent, is necessary to improve the selectivity toward the cyclopropanation reaction. Using the sterically hindered chiral Rh_2_(*R*-PTAD)_4_ dimer, Davies et al. obtained excellent enantioselectivities (up to 99:1 er) when the reaction was run in the presence of an oxidant to generate the diazoalkane in situ. Independently, Ghanem et al. disclosed the asymmetric synthesis of trifluoromethyl cyclopropanes through the highly enantioselective [Rh_2_(*S*-*tert*PTTL)_4_]-catalyzed cyclopropanation reaction of styrene with 2,2,2-trifluoromethyl-1-phenyl-1-diazoethane (Scheme 2).^43^ This new catalyst, [Rh_2_(*S*-*tert*PTTL)_4_], derived from an (*S*)-amino acid is more synthetically accessible compared with [Rh_2_(*S*-PTAD)_4_] previously used by Davies et al. The reaction of the reagents in the presence of 2 mol % of the catalyst in TFT at room temperature led to the cyclopropanes in 99% yield and high enantioselectivity. This method was successfully applied to α-diazophosphonate esters and α-phenyl-α-diazoacetonitrile.^43^

**Scheme 3:**
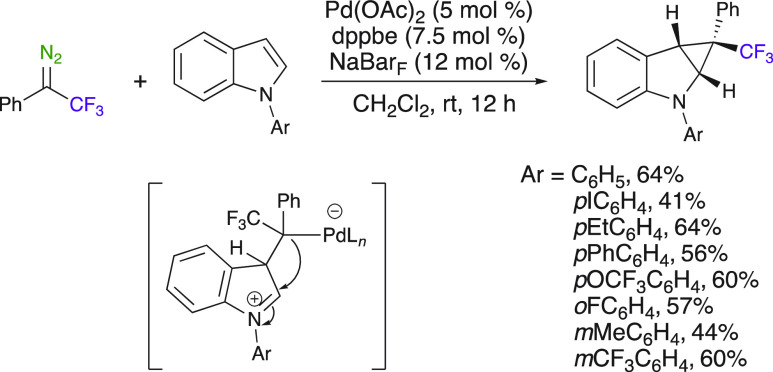
Pd-Catalyzed Cyclopropanation of *N*-Aryl Indoles

Palladium was also used as a metal catalyst in cyclopropanation reactions. In 2020, Koenigs et al. disclosed the synthesis of trifluoromethyl-substituted cyclopropanes from the reaction of aryl trifluoromethyl diazo compounds with *N*-aryl indoles in the presence of a palladium catalyst in mild conditions (Scheme 3).^44^ This method affords trifluoromethyl-substituted *N*-aryl indoles in moderate yields. While the indole can be protected with various aryl groups, the Boc- or Piv-protected indoles did not lead to the cyclopropanes when using the same conditions. It is interesting to notice that the chemoselectivity of the transformation was completely switched when using *N*-methylindole. In this case, a difluoroalkene was obtained instead of the cyclopropane (Scheme 20). The selectivity toward cyclopropanation was rationalized by the lower nucleophilicity of the *N*-aryl indole heterocycles versus that of *N*-methyl. Notably, these two reaction pathways can be swapped from one to another simply by the nature of the group on the indole nitrogen. Indeed, the same aryl trifluoromethyl diazo compounds will lead to divergent reaction outcomes.

**Scheme 4:**
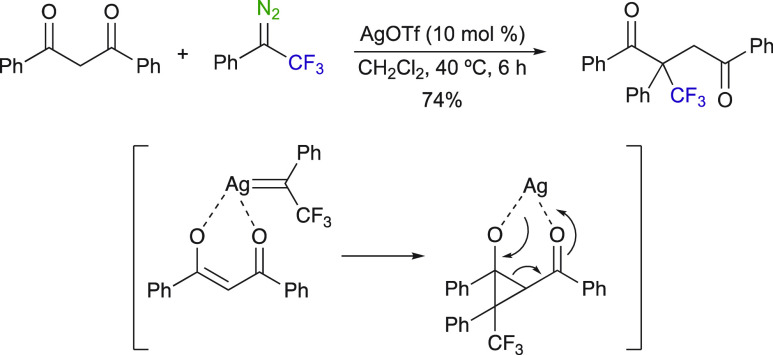
AgOTf-Catalyzed C–C Bond Formation via the Cyclopropanation of an Enolizable 1,3-Dicarbonyl Compound

Cyclopropanation reactions can also be conceivable with enolizable 1,3-dicarbonyl compounds. Bi et al. was able to highlight the ease of forming new C–C bonds using 1,3-dicarbonylated substrates from aryl trifluoromethyl diazo compounds (Scheme 4).^45^ The coordination of the enolizable 1,3-dicarbonyl with the in situ generated Ag^I^-carbene leads to an intermediate cyclopropane, which undergoes a retro-aldol reaction to afford a 1,4-dicarbonyl compound in a 74% yield. This reaction is basically a formal insertion into a C–C bond of 1,3-dicarbonyl compounds. Interestingly, when AgOTf was replaced with Sc(OTf)_3_ as a catalyst, a different chemoselectivity was observed, and in this case, the C–H insertion was obtained instead. This highly efficient catalyst-controlled chemoselectivity accounts for the extraordinary versatility of diazoalkanes.

**Scheme 5:**
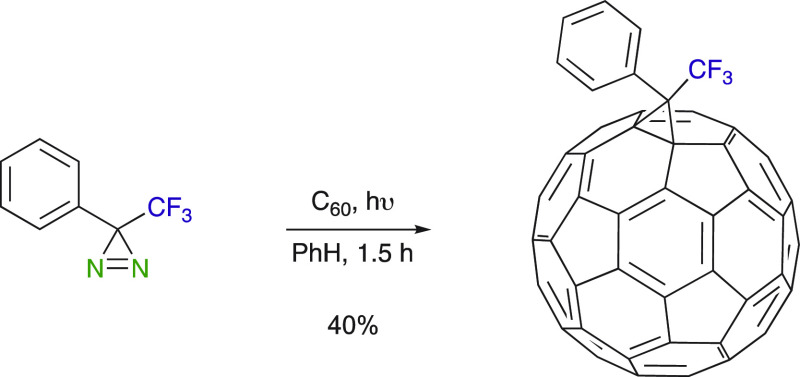
Functionalization of Fullerene C_60_ with Phenyl Trifluoromethyl Diazirine

Although trifluoromethyl diazirines have been employed as photoaffinity reagents to label receptors and linking reagents,^9,17−24^ aryl trifluoromethyl diazirines also found use in the functionalization of fullerenes, that is, as a fluorine tag of C_60_ (Scheme 5).^46^ Whereas diazirines can react with fullerenes via diazoalkanes and/or carbene precursors to give the corresponding [5,6] and [6,6] adducts,^47,48^ the formation of [5,6] open fulleroid was seen to be negligible from phenyl trifluoromethyl diazirine, suggesting that its photolysis yielded only carbene as the intermediate. The formation of the [6,6] adduct to C_60_ occurred, as evidenced by the typical visible absorption spectrum of the product. This photolabeling method aroused great hope for the preparation of new biofunctionalized fullerenes.

#### Cyclopropenations

**Scheme 6:**
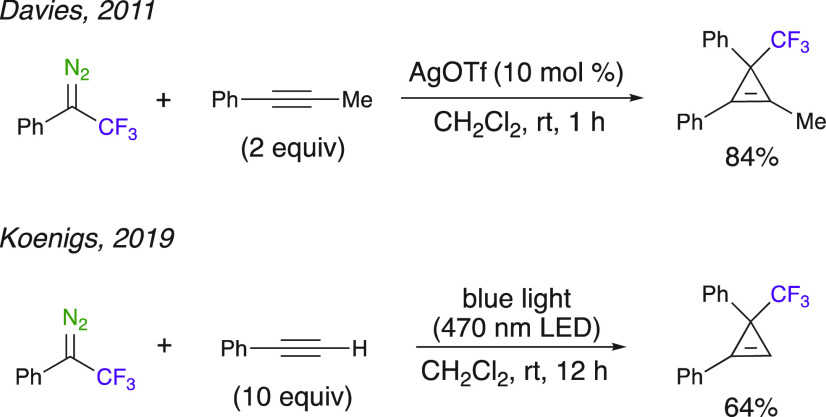
Catalyst-Free Photochemical Synthesis of Substituted Cyclopropenes

Efficient metal-catalyzed cyclopropenation reactions of 2,2,2-trifluoromethyl-1-phenyl-1-diazoethane have been described, including asymmetric versions using chiral Ir^I^ and Rh^II^ complexes. Yet, only recently have metal-free light-mediated methods been established to prepare trifluoromethyl-substituted cyclopropenes. Davies et al. developed a new method for the synthesis of cyclopropenes of internal alkynes using donor/acceptor-substituted diazo compounds using a silver salt in mild conditions.^49^ Using a trifluoromethyl group as an electron-withdrawing substituent on the diazoalkane substrate was also possible as the trifluoromethyl-substituted cyclopropene was formed in good yield (84%, Scheme 6). (1-(Trifluoromethyl)cycloprop-2-ene-1,2-diyl)dibenzene was synthesized through a catalyst-free photochemical carbene transfer reaction of 2,2,2-trifluoromethyl-1-phenyl-1-diazoethane with phenylacetylene. This visible-light-mediated reaction was carried out in a practical way without the need to exclude moisture and air.^50^

**Scheme 7:**
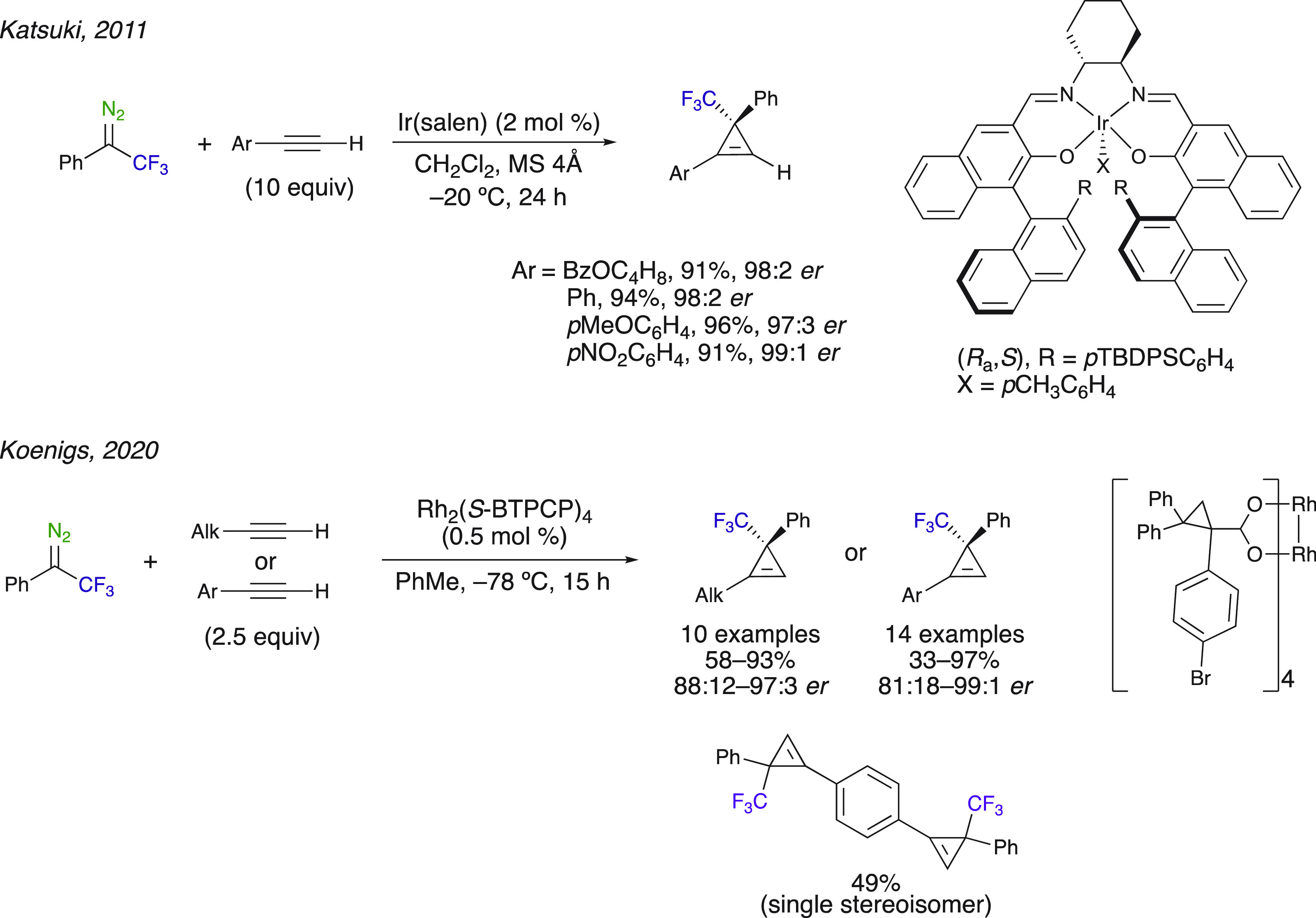
Asymmetric Cyclopropenation of Trifluoromethyl Diazoalkanes with Alkynes

Asymmetric [2 + 1] cyclopropenation reactions were also disclosed from 2,2,2-trifluoromethyl-1-phenyl-1-diazoethane. Katsuki et al. used a chiral iridium(III) complex(salen) for the asymmetric synthesis of trifluoromethylated cyclopropenes (Scheme 7).^51^ Highly enantioselective cyclopropenation was obtained using 2,2,2-trifluoro-1-phenyl-1-diazoethane and various monosubstituted aryl alkynes. Excellent results were also obtained with a rhodium catalyst on a variety of substrates.^52^ Chiral CF_3_-cyclopropenes and oligocyclopropenes were synthesized through the reaction of trifluoromethyl-substituted donor–acceptor diazoalkanes with aliphatic and aromatic terminal alkynes in the presence of commercially available Rh^II^ catalysts, such as Rh_2_((*S*)-BTCP)_4_. It is noteworthy to mention that the catalyst loading is rather low (i.e., 0.5 mol %), and only a small excess of the alkyne was needed. By running the reaction at 0 °C with 1,4-bis(ethynyl)benzene, Koenigs et al. obtained bis-cyclopropenes as the only products over the monocyclopropane intermediates. The outstanding advantage of the method is the very low catalyst loading used, allowing excellent enantioselectivities.

**Scheme 8:**
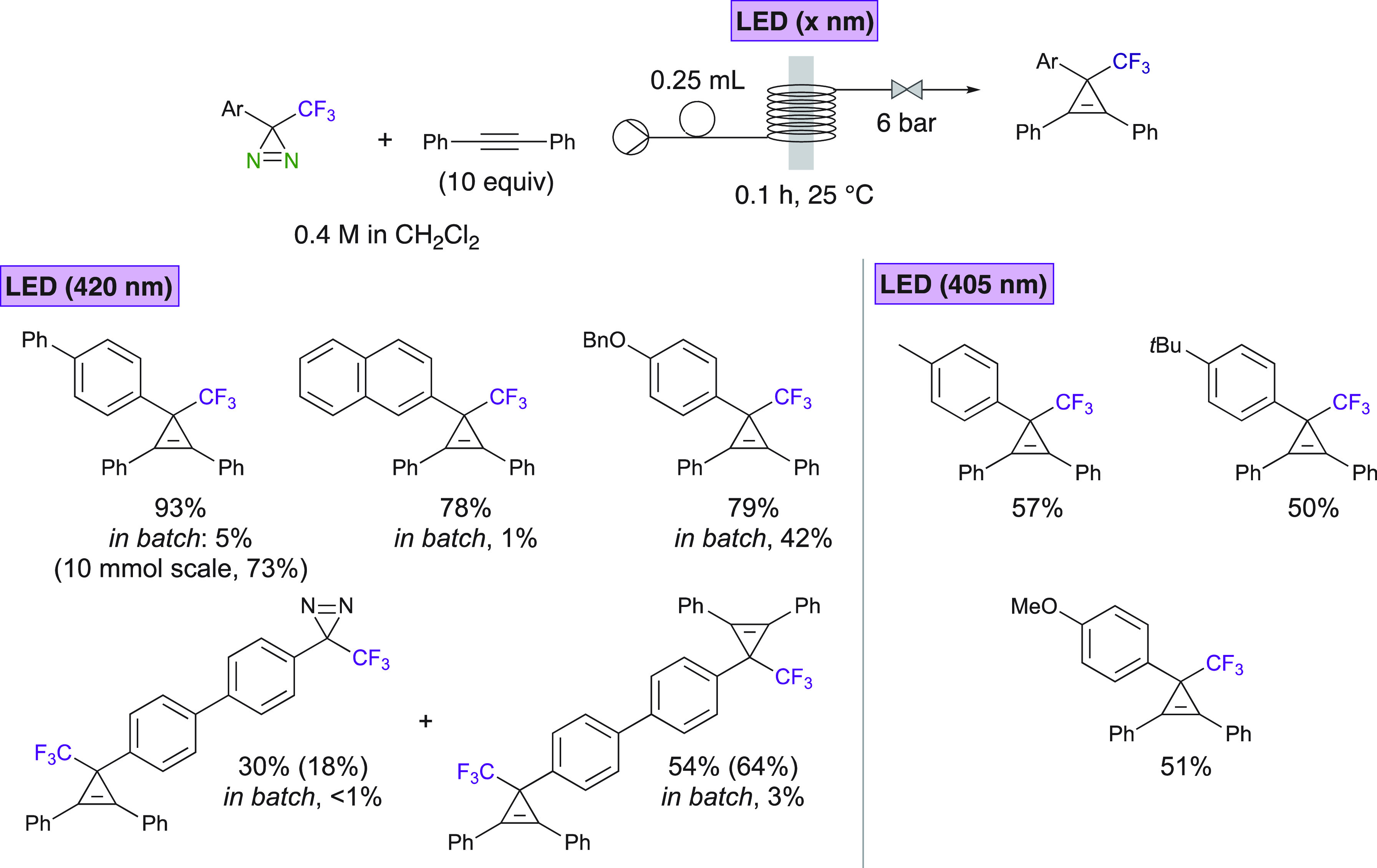
Asymmetric Cyclopropenation of Trifluoromethyl Diazo Compounds with Alkynes in Continuous Flow

Combining photochemical methods and continuous flow chemistry attracted a lot of attention. 3-Trifluoromethyl-3-arylcyclopropenes were obtained by Ollevier et al. via the [2 + 1] cycloaddition of alkynes with photochemically generated carbenes from diazirines (Scheme 8).^42^ This reaction was run in continuous flow using readily available LEDs in mild reaction conditions. The isolated yields were systematically higher than those obtained when running the reaction in batch conditions.

**Scheme 9:**
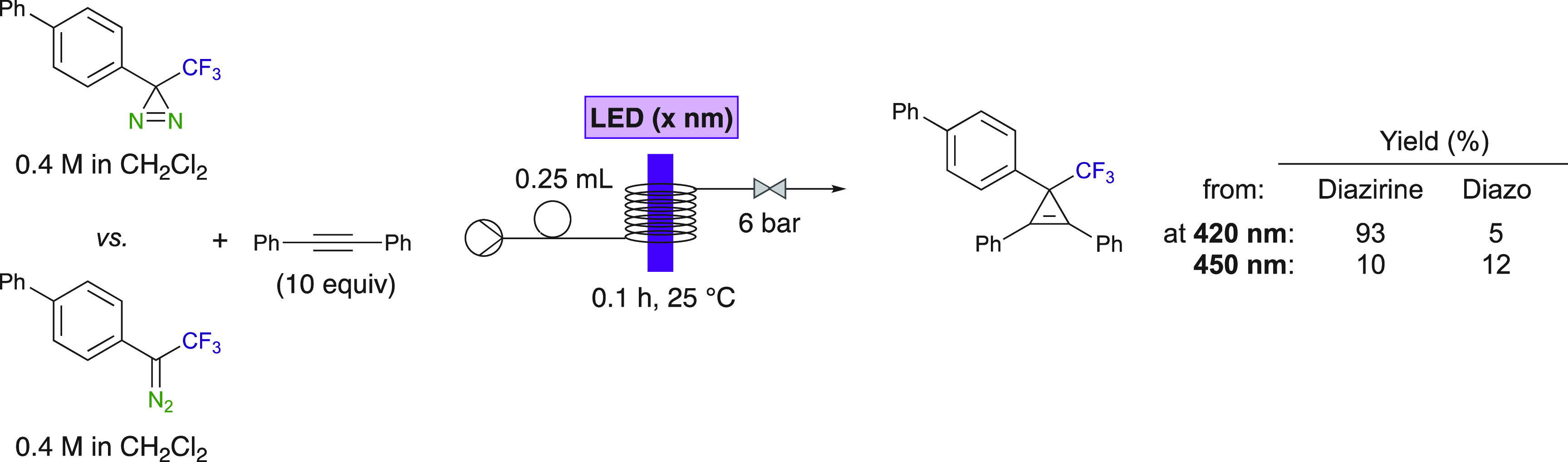
Comparison between Diazirine versus Diazo Compound for Cyclopropenation Reaction in Continuous Flow

Interestingly, control experiments demonstrated that diazirines are more efficient than diazoalkanes when reacted with diphenylacetylene under the same reaction conditions (Scheme 9). It was observed by Ollevier et al. that the cyclopropene was obtained in an excellent yield from the diazirine (93%) when using a 420 nm LED. The use of LED of the visible light spectrum (i.e., purple LED) is also a major asset. When lowering the energy of the light to better match the diazoalkane absorption, the yields of the cyclopropene from the diazoalkane never reached the ones obtained from the diazirine at 420 nm. Thus, diazirines are, by far, better substrates for accessing diaryl trifluoromethyl cyclopropenes by LED irradiation. Noteworthy, isomerization of the diazirine into the diazoalkane takes place to some extent, as demonstrated by in situ IR studies.^42^

### [2 + 3] Cycloadditions

**Scheme 10:**
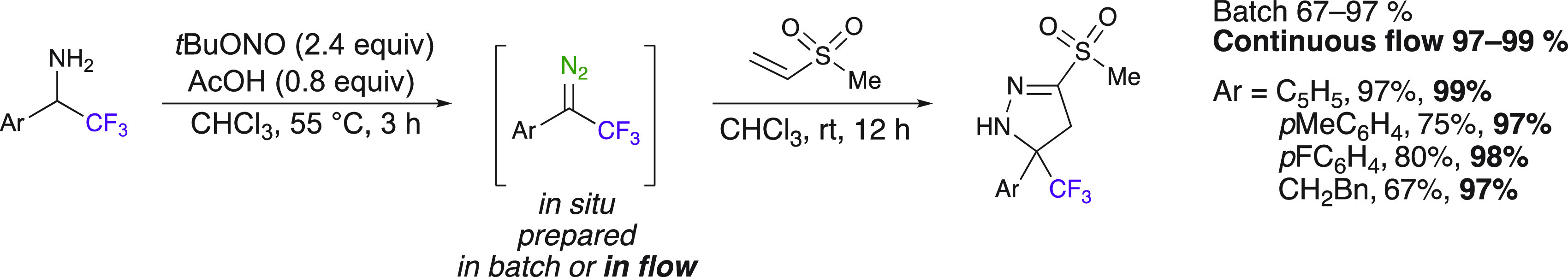
[2 + 3] Cycloaddition Reaction with Vinyl Sulfones for the Synthesis of Pyrazolines

The use of a continuous flow setup to make the trifluoromethyl diazoalkane in situ was also a major asset in [2 + 3] cycloaddition. The synthesis of valuable fluoroalkyl- and sulfone-substituted pyrazolines was reported by Koenigs et al. through the [2 + 3] cycloaddition reaction of fluorinated donor–acceptor diazo compounds with vinyl sulfones (Scheme 10).^53^ Various donor–acceptor fluorinated diazoalkanes were prepared in situ by amine diazotization using either batch or flow conditions. The latter ones provided even better yields of pyrazolines under safer conditions. The opportunity of preparing the aryl trifluoromethyl diazoalkane in situ will clearly open future avenues.

### Multicomponent Reactions

The use of trifluoromethyl diazoalkanes could be effectively promoted in a few elegant multicomponent reactions. The next examples demonstrate the outstanding chemoselectivities obtained in Rh^II^- and Cu^I^-catalyzed multicomponent reactions and the challenging access to selected trifluoromethyl-substituted heterocycles.

### Staudinger Reaction: Formal [2 + 2] Cycloaddition

**Scheme 11:**
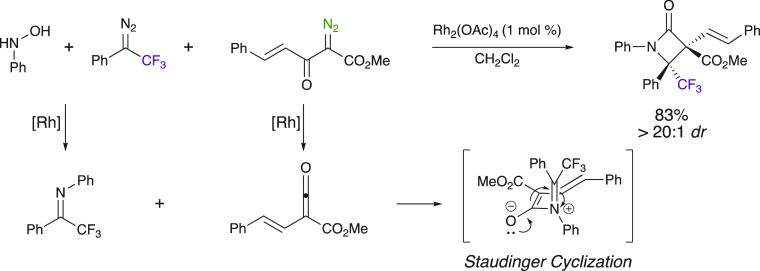
Rh^II^-Catalyzed β-Lactam Synthesis through Formal [2 + 2] Cycloaddition of an Imine with a Ketene

An example of the synthesis of β-lactams using trifluoromethyl diazo compounds was disclosed by Zhang et al.^54^ In this approach, methyl-(2*R*,3*S*)-4-oxo-1,2-diphenyl-3-((*E*)-styryl)-2-(trifluoromethyl)azetidine-3-carboxylate was synthesized through a three-component reaction of *N*-hydroxyaniline, 2,2,2-trifluoro-1-phenyl-1-diazoethane, and methyl-2-diazo-3-oxo-5-phenylpent-4-enoate with good yield and excellent diastereoselectivity (Scheme 11). The transformation was initiated by the Rh^II^-catalyzed imine formation from the reaction of hydroxylamine and the aryl trifluoromethyl diazoalkane, together with the Wolff rearrangement of methyl-2-diazo-3-oxo-5-phenylpent-4-enoate. Thermal Staudinger cyclization of the imine with the ketene intermediate obtained from the Wolff rearrangement gave highly functionalized fluorinated β-lactams in a highly stereoselective manner.

**Scheme 12:**
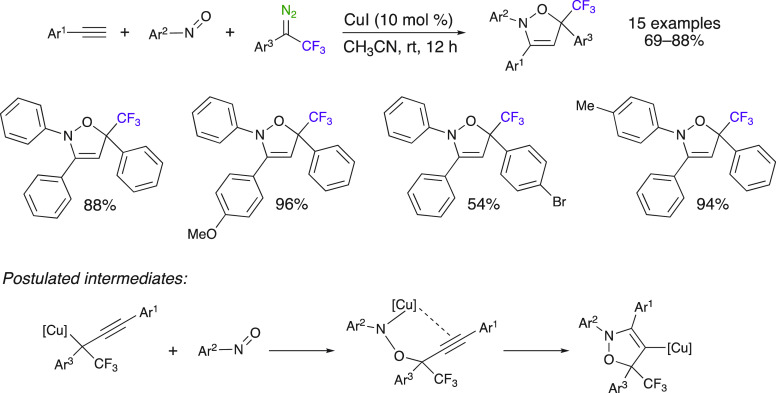
[1 + 2 + 2] Cyclization via a Three-Component Reaction

Trifluoromethyl diazo compounds can also be used for the synthesis of five-membered rings via [1 + 2 + 2] cycloaddition (Scheme 12).^55^ This method developed by Hu and Xing et al. is based on a copper(I)-catalyzed three-component reaction between terminal alkynes, nitrosobenzenes, and aryl trifluoromethyl diazoalkanes. Excellent yields (69–88%) of the trifluoromethyl dihydroisoxazoles were obtained under mild conditions. The mechanism of the reaction involves the electrophilic trapping of the nitrosobenzene by a copper carbene species generated by the reaction of the alkyne with the in situ generated copper carbene species. This method is a great example of the high chemical diversity achieved through multicomponent reactions.

**Scheme 13:**
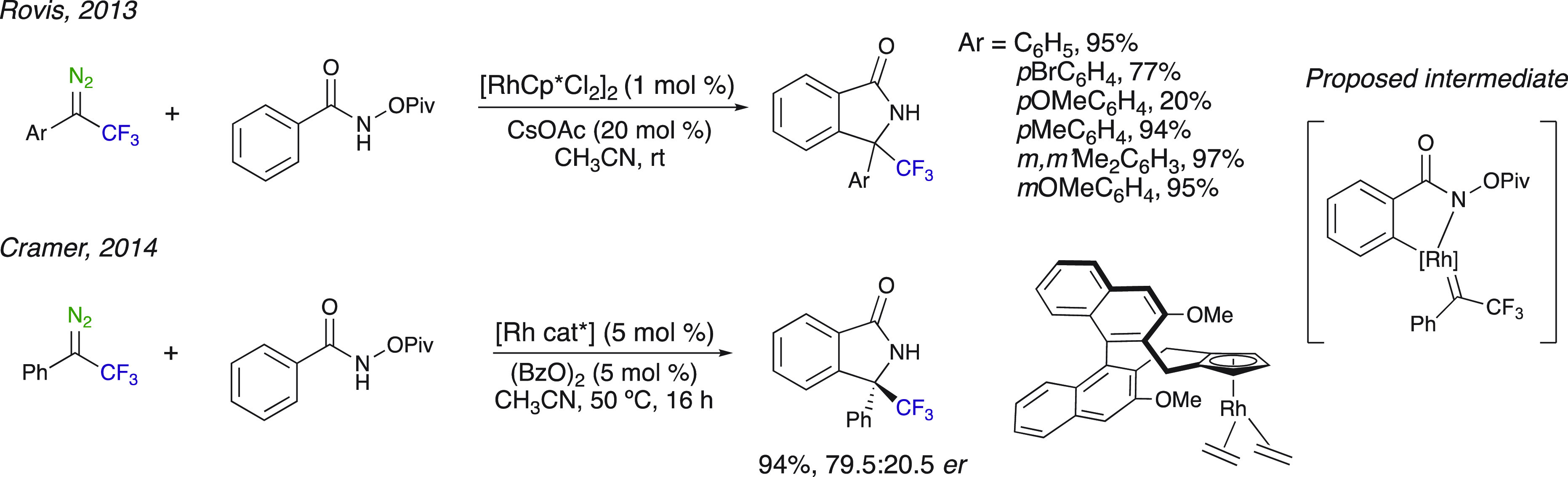
Rh(III)-Catalyzed Synthesis of Trifluomethylated Isoindolones

Rhodium(III) catalysis for the synthesis of isoindolones also involved diazo compounds. Rovis et al. was the first to develop this method for the synthesis of six trifluomethylated isoindolones in 77–97% yields (Scheme 13).^56^ These products possess a quaternary carbon substituted with a trifluoromethyl group and an aryl group, which is a challenging motif to access. The authors provided control experiments suggesting that the C–H activation is turnover-limiting and irreversible. Electron-deficient aromatic groups on the diazoalkane substrates afforded high yields of product, whereas electron-rich aromatic groups had a detrimental effect on the yields. An enantioselective method was developed by Cramer et al., who used a chiral Rh^III^ catalyst comprising a Cp ligand with an atropochiral biaryl backbone giving access to a wide range of enantioenriched chiral isoindolones with high enantioselectivities (Scheme 13).^57^ A trifluoromethylated isoindolone was obtained in an excellent 94% yield, albeit with a lower enantioselectivity (79.5:20.5 er). The proposed intermediate undergoes two insertion steps on the metal carbene. The first step, which is the enantioselective event determining one, is the insertion of the Ar group on the metal carbene, followed by the insertion of the N–H bond generating the corresponding isoindolone.

## Insertion Reactions

Insertion reactions have been extensively used in diazoalkane chemistry. Their applications in aryl trifluoromethyl diazo compounds have been studied in various X–H bonds.

### Insertion Reactions into X–H Bonds (X = B, Si, Sn)

**Scheme 14:**
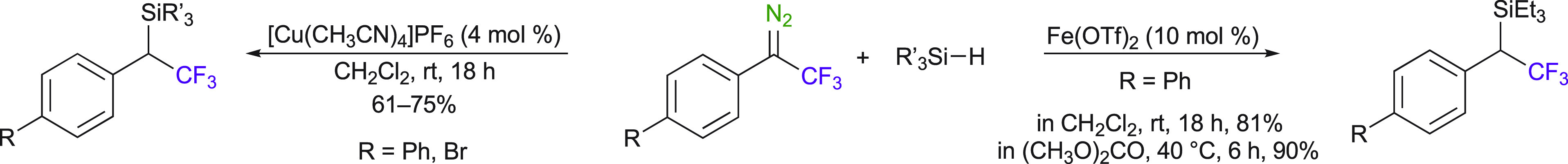
Cu^I^- and Fe^II^-Catalyzed Insertion Reactions into the Si–H and B–H Bonds

The use of a Cu^I^ salt as a catalyst for mediating the decomposition of trifluoromethyl diazo compounds has been exploited in the insertion reaction into X–H bonds. In particular, Gouverneur et al. developed a method suitable for the insertion into Si–H bonds (Scheme 14).^58^ The insertion of a broad range of diazo compounds to Si–H bonds by an Fe^II^ complex in dimethyl carbonate as an emerging green solvent was developed by Ollevier et al. Good to excellent yields of organosilanes were obtained (75–90%, Scheme 14).^59,60^ The use of this solvent was proved by the same authors to be beneficial in a Cu^I^-catalyzed asymmetric version of this reaction (Scheme 16).

**Scheme 15:**
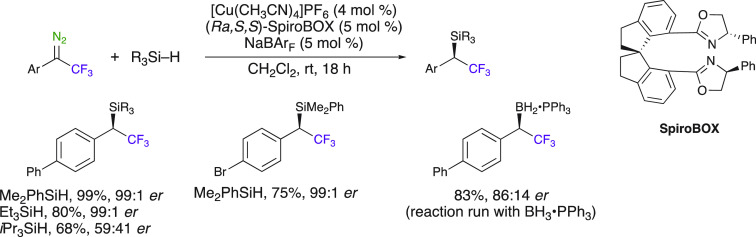
Asymmetric Cu(I)-SpiroBOX-Catalyzed Insertion Reactions into Si–H and B–H

An asymmetric version of the Cu^I^-catalyzed insertion reaction was developed by Gouverneur et al., allowing the synthesis of enantioenriched organosilanes and organoboranes (up to 98% ee) (Scheme 15). Excellent isolated yields and enantioselectivities up to 99:1 were reported for the X–H (X = Si, B) insertion reactions of 2,2,2-trifluoro-1-phenyl-1-diazoethanes using the Cu^I^ salt Cu(CH_3_CN)_4_PF_6_ coordinated with a chiral spiro ligand. However, the expensive multistep synthesis of spiroBOX ligands might be detrimental to the generality of the method.

**Scheme 16:**
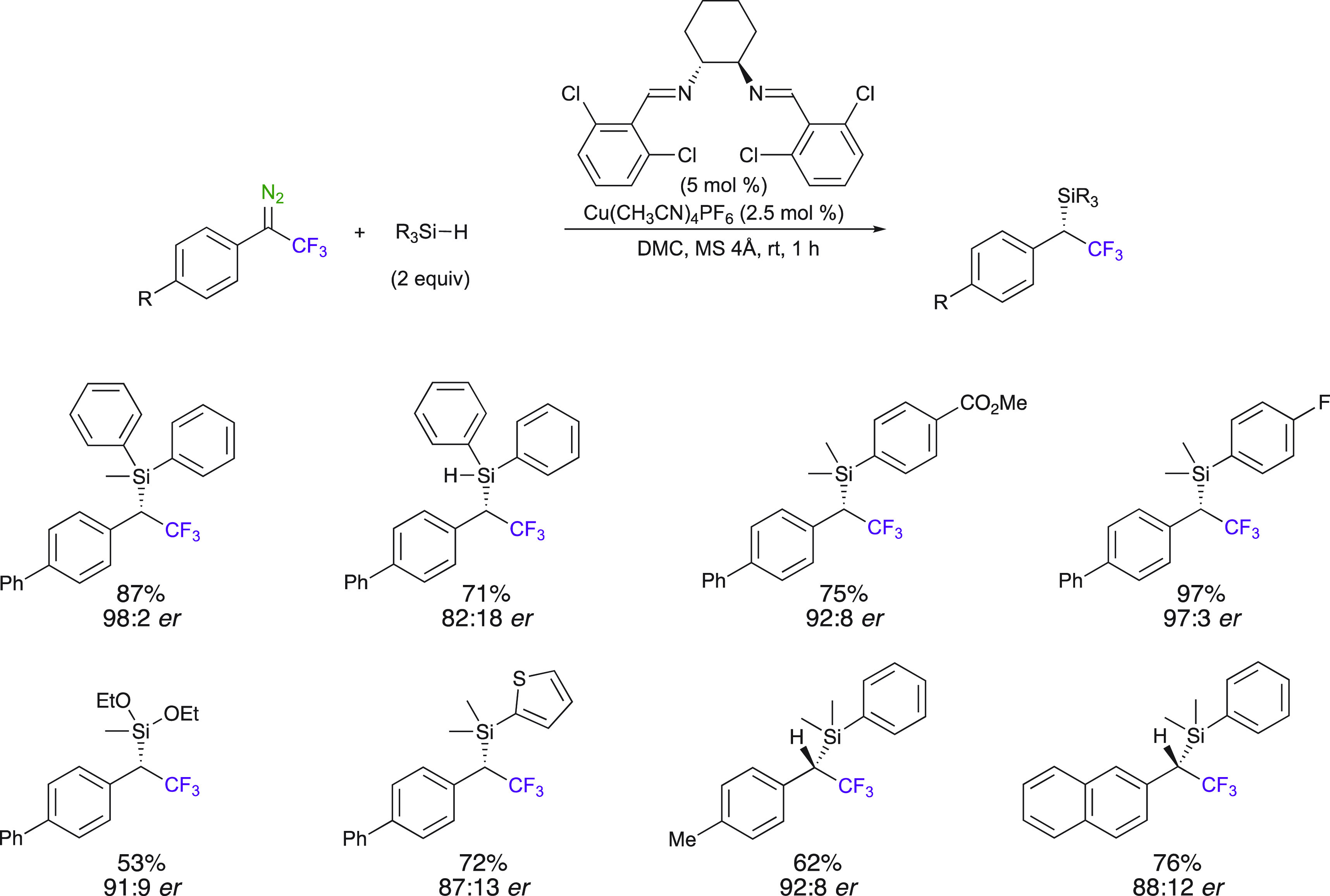
Asymmetric Cu^I^–Catalyzed Insertion Reactions into Si–H Using Bis((2,6-Dichlorobenzylidene)diimino)cyclohexane Ligand

Asymmetric copper(I)-catalyzed Si–H insertion of 2,2,2-trifluoro-1-phenyl-1-diazoethanes in dimethyl carbonate (DMC) was reported by Ollevier et al. (Scheme 16).^41^ A simple and practical Cu^I^ bis((2,6-dichlorobenzylidene)diimino)cyclohexane catalyst for the Si–H insertion reaction of 1-aryl-2,2,2-trifluoro-1-diazoethanes gave yields up to 98% and er values up to 98:2. In these conditions, high enantioselectivities were obtained at room temperature. An important aspect is also that the ligand is easily prepared in two steps. The method is applicable to aryl trifluoromethyl diazo substrates and to a large variety of organosilanes.

**Scheme 17:**
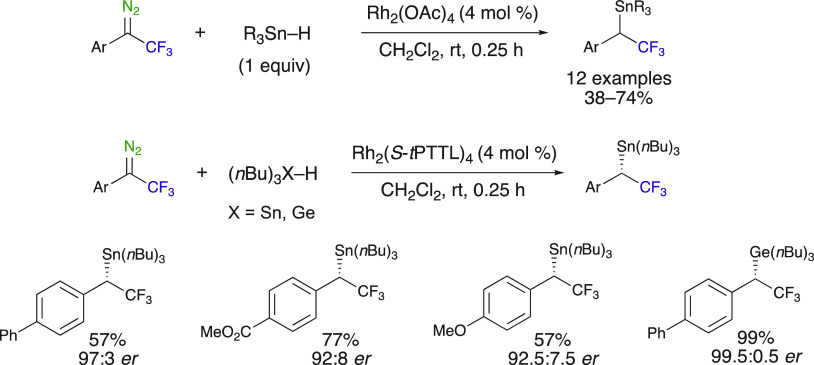
Rh^II^-Catalyzed Insertion Reactions into Sn–H

The insertion of trifluoromethyl diazo compounds into the Sn–H bond was demonstrated by Gouverneur et al. through the use of a rhodium(II) catalyst, where Cu(CH_3_CN)_4_PF_6_ appears to be ineffective.^61^ This method was applied on various trifluoromethyl diazoalkanes, and moderate to good yields were reported using Rh_2_(OAc)_4_ as the catalyst (12 examples, 38–74%, Scheme 17). The asymmetric Rh^II^-catalyzed insertion reaction of 1-aryl-substituted 2,2,2-trifluoro-1-diazoethanes into tin hydrides was also developed using Rh_2_((*S*)-*t*PTTL)_4_ (Scheme 16). Delivering corresponding enantioenriched α-(trifluoromethyl)benzyl stannanes, this method is in contrast with diazo esters, which mainly afford CH_2_ reduction products. Asymmetric insertion reactions into the Ge–H and the Si–H bonds were also reported with good to excellent yields and 99% ee for both of them using the same Rh_2_((*S*)-*t*PTTL)_4_. Notably, an α-(trifluoromethyl)benzyl germane could be obtained in an excellent enantioselectivity.

**Scheme 18:**
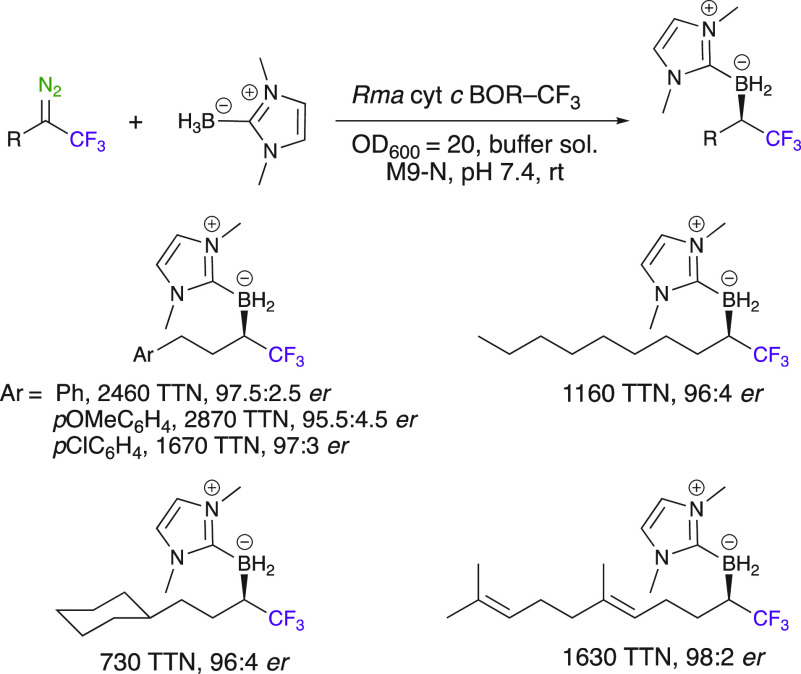
Chiral Organoboron Synthesis via Enzymatic Directed Evolution

The interest of aryl trifluorodiazoalkanes was also very nicely demonstrated in biocatalysis. Arnold et al. in 2019 developed a biocatalytic platform by reprogramming heme proteins of *Rhodothermus marinus* cytochrome *c* (Rma cyt *c*) to utilize trifluorodiazoalkanes for highly enantioselective carbene B–H insertion reactions (Scheme 18).^62,63^ This enzymatic engineering method—enzymatic directed evolution—allows one to selectively control the enantioselectivity of an enzyme. The developed system using the *Rma* cyt *c*BOR–CF_3_ enzyme can accept a broad range of trifluorodiazoalkanes to produce chiral versatile α-trifluoromethylated (α-CF_3_) boranes with turnovers up to 2460 and enantiomeric ratios up to 98.5:1.5. The enantiopreference of the biocatalyst could be tuned to provide either enantiomer of the organoborane products. Stereospecific transformation of these synthetic building blocks was also demonstrated, for example, the synthesis of the boronic acid from the borane with retention of configuration.^63^

### Insertion Reactions into N–H, P–H, O–H, and S–H Bonds

**Scheme 19:**
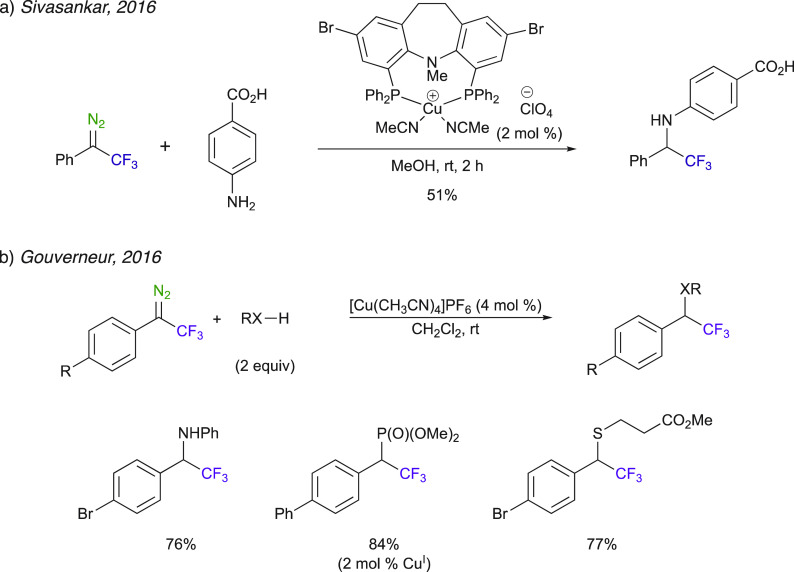
Cu^I^-Catalyzed Insertion Reactions into the N–H, P–H, and S–H Bonds

Whereas the insertion reactions into N–H, P–H, O–H, and S–H bonds can vary from the insertion reactions disclosed in the previous section in a mechanistic perspective, trifluoromethyl diazo compounds have also been used in insertion reactions into the X–H where X is a heteroatom. The use of copper(I) as a catalyst for the decomposition of aryl trifluoromethyl diazo compounds has often been exploited in numerous insertion examples. The chemoselective carbene insertion into the −NH bond over −COOH bonds was described by Sivasankar et al. using a phosphine-ligand-stabilized air-stable Cu^I^ complex (Scheme 19a).^64^ Gouverneur et al.’s method for the insertion reaction of diazo compounds into the Si–H and B–H bonds (Scheme 14) was also extended to the insertion into N–H, P–H, and S–H bonds (Scheme 19b).^58^

### Insertion Reactions into C–H Bonds

**Scheme 20:**
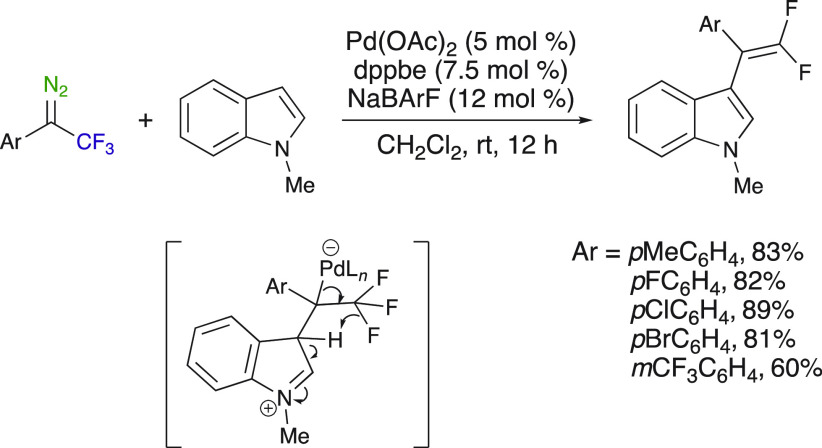
Pd-Catalyzed Introduction of a (2,2-Difluorovinyl)benzene Group onto *N*-Alkylindole

The insertion reactions into C–H bonds have been often used with aryl trifluoromethyl diazirines in chemical biology applications—as pointed to in the Introduction. This type of insertion reactions involving aryl trifluoromethyl diazoalkanes has admittedly been less studied. Major advancements have been made using palladium and copper catalysts. Koenigs et al. disclosed the Pd^II^-catalyzed introduction of a (2,2-difluorovinyl)benzene group onto *N*-alkylindole heterocycles through carbene transfer of fluorinated diazoalkanes under the careful choice of ligand and mild conditions. This process involved a palladium-catalyzed C–H functionalization followed by a β-fluoride elimination reaction between an *N*-methylindole and an aryl trifluoromethyl diazo compound (Scheme 20).^44^ As mentioned previously, *N*-aryl indoles led to the cyclopropanation reaction instead of the β-fluoride elimination reaction under the same reaction conditions (Scheme 3). Appreciably, only substituting the group on the indole N resulted in a different reaction pathway.

**Scheme 21:**
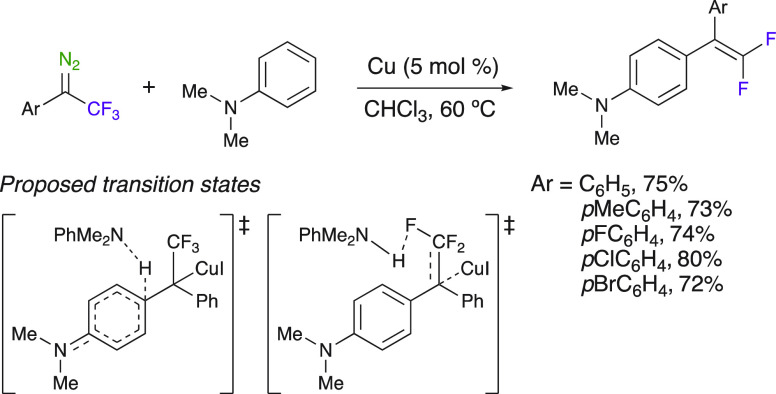
C–H Functionalization of Anilines and *gem*-Difluoroalkene Synthesis

A simple copper-catalyzed and one-step *gem*-difluoro alkenylation reaction of electron-rich aniline derivatives with fluorinated diazo compounds via C–H functionalization was further developed by Koenigs et al. (Scheme 21).^65^ This highly chemoselective reaction occurred via the nucleophilic attack of the aniline derivatives to an electrophilic copper carbene complex, followed by a second molecule of aniline used as a basic agent for facilitating the elimination of HF, therefore demonstrating the dual role of aniline. This method allows the synthesis of *gem*-difluoroalkenes with excellent yields and was also extended to indolines and tetrahydroquinolines.

**Scheme 22:**
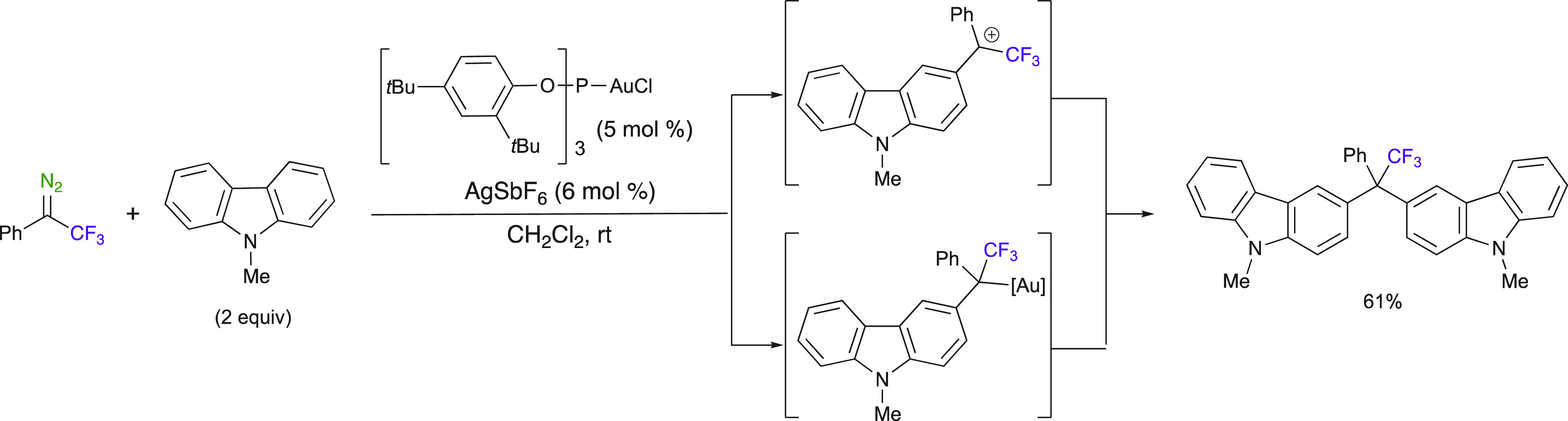
Au^I^-Catalyzed C–H Functionalization Reaction of *N*-Methyl Carbazole

The multiple C–H functionalization reaction of *N*-methyl carbazole with trifluoromethyl phenyl diazo was also disclosed by the same author (Scheme 22).^66^ Double C–H functionalization arose from the trifluoromethyl diazo compound and 2 equiv of *N*-methyl carbazole, affording the trifluoromethyl phenyl–alkyl moiety to be linked with two *N*-methyl carbazoles. The reaction was believed to occur through a Friedel–Crafts-type electrophilic substitution reaction catalyzed by the phosphite-derived Au^I^ complex. This is the only report to date of a Au^I^ carbene intermediate obtained from 2,2,2-trifluoromethyl-1-phenyl-1-diazoethane. Gold catalysts are likely to arise as very promising ones used in carbene chemistry.

**Scheme 23:**
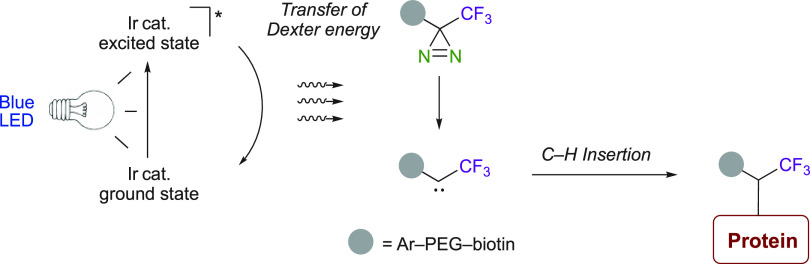
Cell Mapping Using Catalytic Sensitization of Trifluoromethyl Diazirines

The photochemical properties of diazirines made them particularly useful as photoaffinity labeling reagents in chemical biology.^23,67^ Indeed, most trifluoromethyl diazirines are decomposed under a 360 nm light irradiation, where a vast range of bioactive molecules do not absorb.^23,24,27,68,69^ The elucidation of protein functions from their structure/activity and the understanding of molecular mechanisms involved in protein–ligand binding is a major challenge in chemical and molecular biology and drug design.^22,24,28,35,36,70−75^ An outstanding example highlighting the use of trifluoromethyl diazirines in chemical biology was disclosed by Fadeyi, Oslund, and MacMillan et al. in microenvironment mapping on immune cells for a better understanding of their role and mode of action (Scheme 23).^29^ Their method uses a biotinylated trifluoromethyl diazirine that will be further conjugated to the studied protein. Using microscopy and observation of cell tags, the mapping on live cells could be obtained. The activation to the excited state of an iridium-based photocatalyst was reached using a blue light.^76^ After fluorescence relaxation and short-range Dexter energy transfer, in which the catalyst is returned to its ground S_0_ state, the energy is transferred to the biotinylated trifluoromethyl diazirines. These diazirines are then decomposed and generate carbenes, leading to C–H insertions with neighboring proteins. This indirect excitation method helps the decomposition of various diazirines without the specific choice of the appropriate LED for each of them. This is the first report on the use of a photocatalyst in the photochemical decomposition of diazirines.

**Scheme 24:**
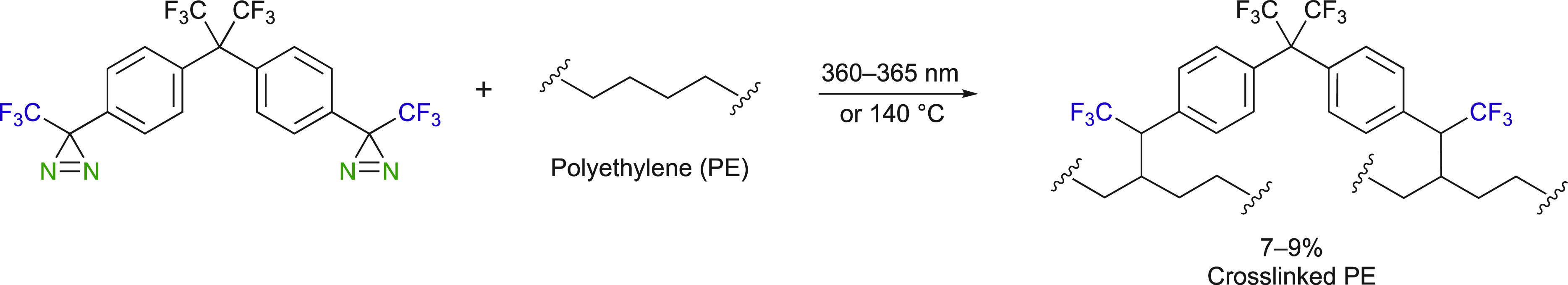
Diazirines as Polymer Cross-Linkers

Trifluoromethyl diazirines have also found promising applications in polymer chemistry and surface chemistry. Polymer chemistry is an attractive field of research in which carbene chemistry can be highly useful. Trifluoromethyl diazirines were used to create cross-linking in a polymer, such as polyethylene.^15,18^ Wulff et al. used the thermal or photochemical decomposition of trifluoromethyl bisdiazirines to perform C–H insertion on polyethylene (Scheme 24). The obtained yields in cross-linked polymers are low, but more importantly, their properties, such as solubility, glass transition temperature (*T*_g_), and resistance, are modified. The higher thermal stability of diazirines versus that of diazo compounds makes them better candidates under the conditions used for cross-linking reactions. Therefore, this method allows the synthesis of cross-linked aliphatic polymers possessing high resistivity, via the C–H insertion run in a controlled manner.

**Scheme 25:**
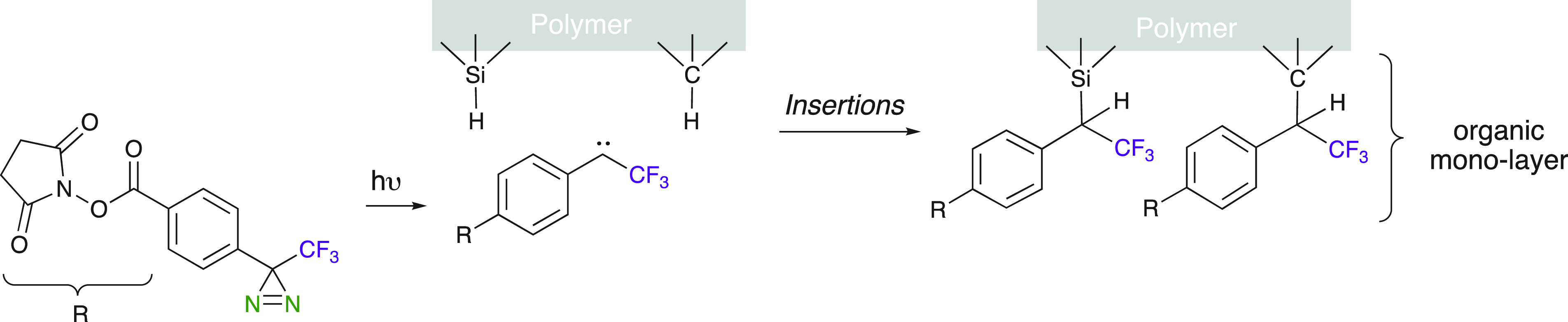
Diazirine Insertion into the Si–H and C–H on a Solid Support

An example of diazirine applications in polymers is their use in monolayers by the insertion into the Si–H and C–H on a solid support (Scheme 25).^77^ This enables one to build additional layers of polymers with different chemical properties (interfacial adhesion, friction, chemical specificity, etc.). Therefore, this functionalization method can be used as a complementary technique as the classical existing SAM (self-assembled monolayer). It can also be applied to substrates which suffer from SAM limitations, such as chemically inert interfaces.

## Coupling Reactions

**Scheme 26:**
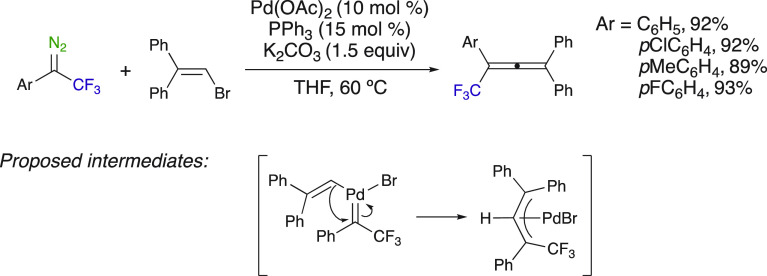
Pd-Catalyzed Synthesis of Tetrasubstituted Trifluoromethyl Allenes from Aryl Diazo Compounds and Vinyl Bromides

Coupling reactions of trifluoromethyl-substituted diazoalkanes provide access to various trifluoromethyl-substituted allenes and alkenes. Koenigs et al. reported the palladium-catalyzed synthesis of trifluoromethyl allenes from vinyl bromides and trifluoromethyl diazo compounds (Scheme 26).^78^ The mechanism of the reaction proceeds via an oxidative addition of a Pd^0^ complex with the vinyl bromide, followed by the addition of the diazoalkane, furnishing a metal carbene. Migration and insertion of the vinyl moiety and insertion, followed by reductive elimination, affords the trifluoromethyl allene. This method was efficient for the synthesis of tetrasubstituted trifluoromethyl allenes up to the gram-scale and under mild conditions. Starting from four different trifluoromethyl diazo compounds, a large variety of symmetrical and nonsymmetrical trifluoromethyl diazo compounds allenes have been disclosed (20 examples).

**Scheme 27:**
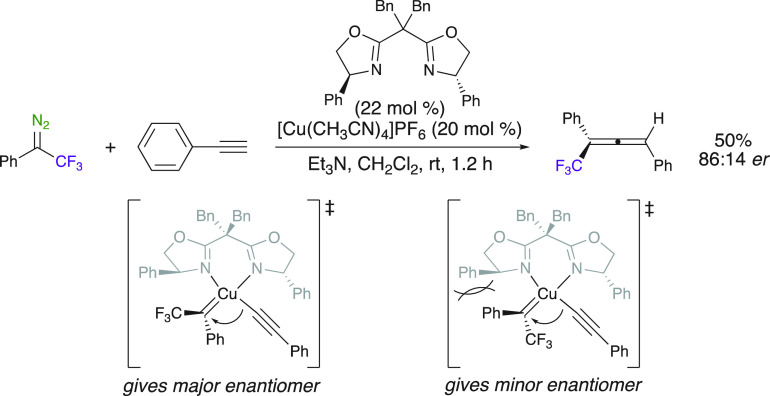
Enantioselective Synthesis of Trisubstituted Trifluoromethyl Allenes with Chiral BOX Ligands

Wang et al. developed an enantioselective synthesis of trisubstituted trifluoromethyl allenes by using a Cu^I^ catalyst with a bisoxazoline (BOX) chiral ligand. In this approach, the key step for the construction of axial chirality is related to alkynyl migratory insertion of Cu^I^ carbene (Scheme 27).^79^ The key step of the enantioselective event is related to the migratory insertion of the alkynyl group. The only example given is insertion of phenyl trifluoromethyl diazo into the C–H bond of phenylacetylene, leading to a 50% yield and a good enantioselectivity (86:14 er). Interestingly, replacement of the CF_3_ group with a CH_3_ led to an increase of yield and stereoselectivity of the trisubstituted trifluoromethyl allenes.

**Scheme 28:**
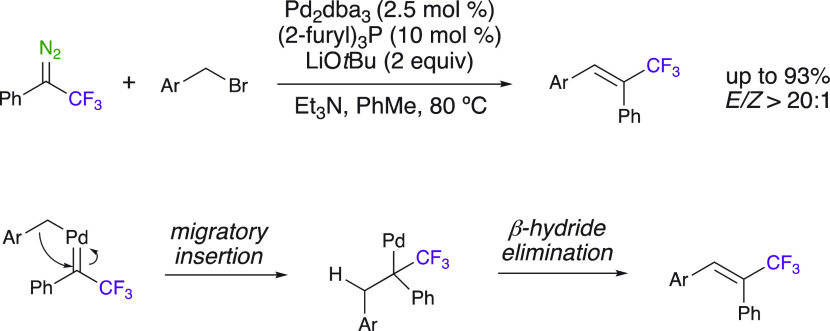
Cross-Coupling Reaction Leading to Trifluoromethylated Olefins

Palladium catalysis applied to trifluoromethyl diazo compounds has been extensively studied by Wang et al.^10^ The use of Pd^II^ mediates both the diazo decomposition and the cross-coupling of the generated Pd-carbene with an aryl bromide, affording the desired trifluoromethylated alkenes after β-hydride elimination (Scheme 28).^80^ A wide range of alkenes have been obtained in moderate to excellent yields (46–93%, 14 examples). This method can also be applied to tosylhydrazones via the in situ generation of the corresponding diazoalkane.

**Scheme 29:**
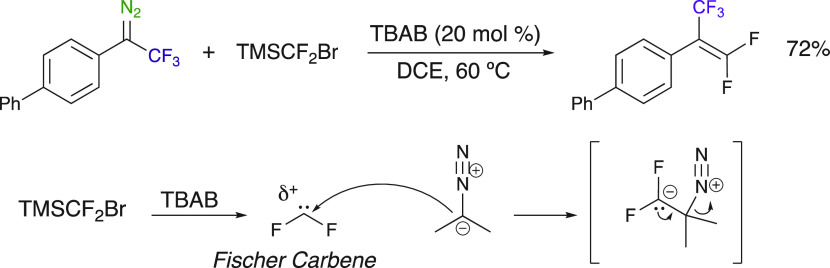
*gem*-Difluoroalkene Synthesis Using TMSCF_2_Br as a Difluoromethylene Source

The direct addition of aryl trifluoromethyl diazo compounds to difluorocarbene allowing the synthesis of disubstituted difluorinated alkenes was reported by Wang et al. (Scheme 29).^81^ The difluorocarbene-type species is generated in situ from the use of TMSCF_2_Br, and a good yield of the polyfluorinated 1,1-difluoroalkene was obtained.

**Scheme 30:**
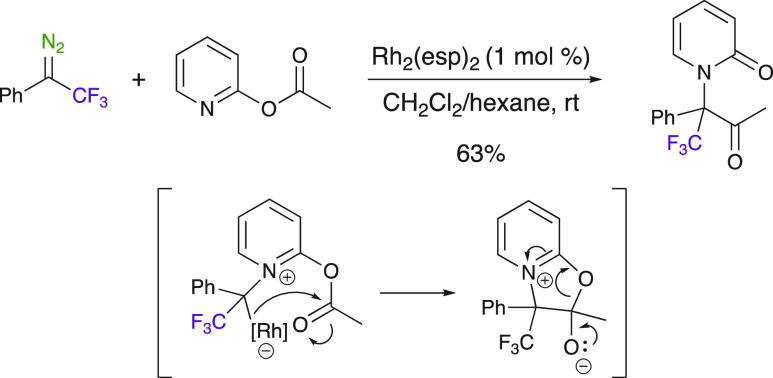
Access to *N*-Substituted Pyridones by Catalytic Intermolecular Dearomatization Followed by 1,4-Acyl Transfer

The dearomatization reaction of 2-pyridones with diazo compounds was reported by Sun and Zhang et al. using various rhodium catalysts (Scheme 30).^82^ The in situ formation of a pyridinium ylide followed by a 1,4-acyl-type rearrangement led to *N*-substituted pyridones. Only one example of a CF_3_-substituted substrate was also reported. An asymmetric variant of the method has also been developed for nonfluorinated substrates using dirhodium(II) tetrakis[*N*-tetrachlorophthaloyl-(*S*)-*tert*-leucinate] as the catalyst.

**Scheme 31:**
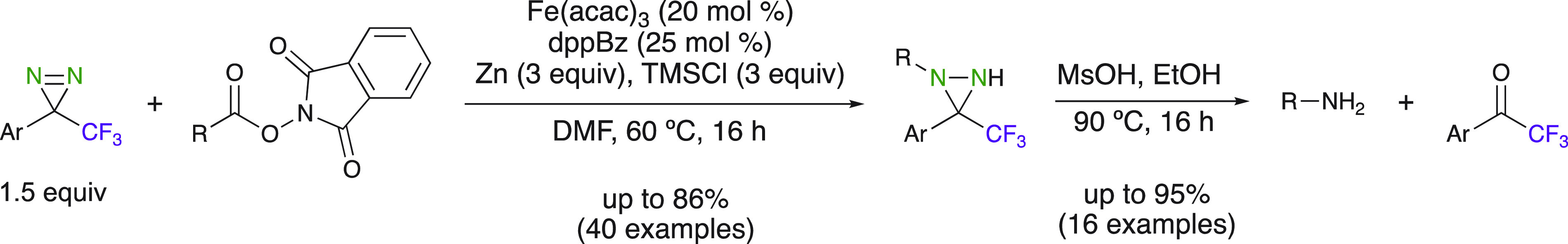
Fe^III^-Catalyzed Decarboxylative Amination of Esters Using Trifluoromethyl Diazirines

While many applications on aryl trifluoromethyl diazirines that involve loss of nitrogen have been reported to date, their transformation into a diaziridine has been investigated to a much lower extent. Lopchuk et al. highlighted the use of diazirines as double electrophilic nitrogen transfer reagents in the decarboxylative amination of esters (Scheme 31).^83^ A series of 50 monosubstituted diaziridines were prepared in one step, giving amines, hydrazines, and nitrogen heterocycles upon acidic treatment. This iron catalytic method (20 mol % of Fe(acac)_3_, 25 mol % of 1,2-bis(diphenylphosphino)benzene dppBz) is an alternative to the other routes involving dioxiranes, aziridiniums, and oxaziridines. Control experiments using TEMPO suggest the involvement of a radical mechanism, the *N*-(acyloxy)phthalimides being used as redox-active esters. A large number of amines were obtained from diverse trifluoromethyl diazirines. This method was further applied to a diazirine bearing a perfluoroalkyl chain (C_8_F_17_) using fluorous phase synthesis.

**Scheme 32:**
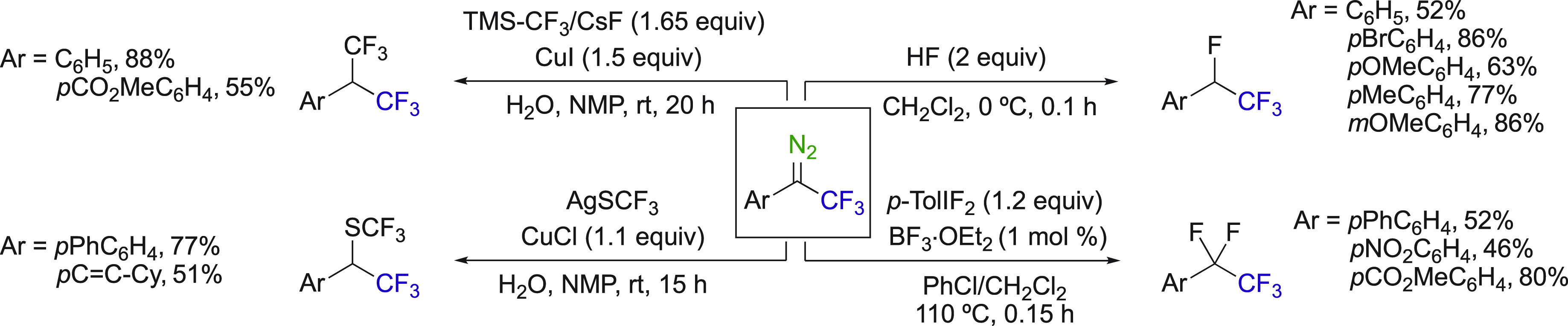
Fluoride Additions on Trifluoromethyl Diazoalkanes

The addition of fluoride and fluoroalkyl-derived groups to aryl trifluoromethyl diazo compounds is an great example for the formation of various fluorinated alkanes (Scheme 32).^84−86^ The efficient trifluoromethylthiolation of diazo compounds through copper-carbene migratory insertion was reported by Wang et al.^85^ Gouverneur et al. notably demonstrated the versatility of the use of these diazo compounds, obtaining excellent yields on a wide range of compounds.^84^ In this specific study, the method of [^18^F]-labeling using [^18^F]fluoride, and not [^18^F]F_2_, was demonstrated for the first time.^84^ These transformations demonstrate that aryl trifluoromethyl diazoalkanes are promising for providing access to a variety of fluorinated building blocks.

## Conclusion and Outlook

The chemistry of diazo compounds continues to be a fascinating part of organic synthesis. Aryl trifluoromethyl diazo compounds appear to be extremely versatile in various synthetic transformations ranging from cycloaddition and insertion reactions to the synthesis of various highly valuable building blocks containing fluorine. The use of diazoalkanes in various reactions is being extended continuously. There is no doubt that there has been increasing interest in synthetic transformations from trifluoromethyl diazo compounds. Numerous reactions have been developed, and these have opened doors to challenging transformations. Trifluoromethyl diazo compounds constitute attractive reactants to develop a wider variety of access to trifluoromethyl-containing molecules and other organofluorine building blocks. Whereas trifluoromethyl diazirines were often used as photoaffinity probes, many synthetic useful applications have now recently appeared in organic synthesis. These results suggest that photochemical decomposition of aryl trifluoromethyl diazirines can now be actively extended to other types of reactions. This family of compounds is definitively a very promising one in synthesis. In particular, the decomposition of trifluoromethyl diazirines using a photocatalyst was only reported by Fadeyi, Oslund, and MacMillan et al. in a chemical biology application and was never reported in synthetic applications. There is clearly a place for new developments using photosentisized decomposition of diazirines. Also, the development of more synthetically useful procedures involving in situ formation of the aryl trifluoromethyl diazoalkanes or the metal-free photochemical formation of the carbene intermediates is also growing rapidly and thus demonstrates the synthetic versatility of aryl trifluoromethyl diazoalkanes. Photochemical reactions run in constant flow have many important assets and will undoubtedly be developed in further applications. In this regard, few articles in coupling reactions have been published yet, whereas the research fields of cycloaddition and insertion reactions have already been much more explored. Undoubtedly, research in coupling chemistry will further expand. Also, the use of more affordable metal catalysts, such as iron salts, remains scarce and should be further developed. Challenging asymmetric reactions via chiral iron carbenes are still in demand. Recent advancements in the chemistry of both trifluoromethyl diazoalkanes and diazirines have led to outstanding achievements toward more efficient synthetic methods. Surely, the current frenetic activity in this field will develop even more and considerably enrich the chemists’ toolbox in fluorine chemistry.
